# An algebraic topological method for multimodal brain networks comparisons

**DOI:** 10.3389/fpsyg.2015.00904

**Published:** 2015-07-06

**Authors:** Tiago Simas, Mario Chavez, Pablo R. Rodriguez, Albert Diaz-Guilera

**Affiliations:** ^1^Departament de Física Fonamental, Facultat de Física, Universitat de BarcelonaBarcelona, Spain; ^2^Department of Psychiatry, University of CambridgeCambridge, UK; ^3^Telefonica Research, Edificio TelefonicaBarcelona, Spain; ^4^Centre National de la Recherche Scientifique-UMR-7225, Hôpital Pitié Salpêtrière, Bat ICMParis, France

**Keywords:** structure-activity relationship, network analysis, functional connectivity, algebraic statistics, multilayer, multiplex

## Abstract

Understanding brain connectivity is one of the most important issues in neuroscience. Nonetheless, connectivity data can reflect either functional relationships of brain activities or anatomical connections between brain areas. Although both representations should be related, this relationship is not straightforward. We have devised a powerful method that allows different operations between networks that share the same set of nodes, by embedding them in a common metric space, enforcing transitivity to the graph topology. Here, we apply this method to construct an aggregated network from a set of functional graphs, each one from a different subject. Once this aggregated functional network is constructed, we use again our method to compare it with the structural connectivity to identify particular brain regions that differ in both modalities (anatomical and functional). Remarkably, these brain regions include functional areas that form part of the classical resting state networks. We conclude that our method -based on the comparison of the aggregated functional network- reveals some emerging features that could not be observed when the comparison is performed with the classical averaged functional network.

## 1. Introduction

In the last decade, the use of advanced tools derived from neuroimaging and complex networks theory have significantly improved our understanding of brain functioning (Sporns, 2011). Notably, connectivity-based methods have had a prominent role in characterizing normal brain organization as well as alterations due to various brain disorders (Varela et al., 2001; Stam and van Straaten, 2012; Stam, 2014). Most of the recent works aim to quantify the role of connectivity in the communication abilities of neural systems. However, the very same notion of connectivity is controversial since data used in brain connectivity studies can reflect functional neural activities (electrical, magnetic or hemodynamic/metabolic) or anatomical properties (Varela et al., 2001; Bullmore and Sporns, 2009). Neuroanatomical connectivity is meant as the description of the physical connections (axonal projections) between two brain sites (Bullmore and Sporns, 2009), whereas functional connectivity is defined as the estimated temporal correlation between spatially distant neurophysiological activities such as electroencephalographic (EEG), magnetoencephalographic (MEG), functional magnetic resonance imaging (fMRI) or positron emission tomography (PET) recordings (Varela et al., 2001).

In recent years, the concept of “brain networks” is becoming fundamental in neuroscience (Stam and Reijneveld, 2007; Bullmore and Sporns, 2009; Stam and van Straaten, 2012; Stam, 2014). Within this framework, nodes stand for different brain regions (e.g., parcelated areas or recording sites) and links indicate either the presence of an *anatomical* path between those regions or a *functional* dependence between their activities. In the last years, this representation of the brain has allowed to visualize and describe its non-trivial topological properties in a compact and objective way. Nowadays, the use of network-based analysis in neuroscience has become essential to quantify brain disfunctions in terms of aberrant reconfiguration of functional brain networks (Stam and Reijneveld, 2007; Stam and van Straaten, 2012; Stam, 2014).

Experimental evidence has revealed, for instance, alterations in functional and anatomical brain networks in normal cognitive processes, across development, and in a wide range of neurological diseases (see Bullmore and Sporns, 2009; Stam, 2014; and references therein). Despite its evident interplay, comparison between anatomical and functional brain networks is not straightforward (Deco et al., 2011; Nicosia et al., 2014). Theoretical studies provide support for the idea that structural networks determine some aspects of functional networks (Deco et al., 2011), but it is less clear how the anatomical connectivity supports or facilitates the emergence of functional networks. Although nodes with similar connection patterns tend to exhibit similar functionality, the functionality of an individual neural node is strongly determined by the pattern of its interconnections with the rest of the network (Nicosia et al., 2014).

Correspondences between functional and structural networks remains thus an active research area (Honey et al., 2007, 2009, 2010). A better understanding of how anatomical scaffolds support functional communication of brain activities is necessary to better understand normal neural processes, as well as to improve identification and prediction of alterations in brain diseases.

In this paper we address this relationship between anatomical and functional connectivity. In previous studies, the correspondence of these networks has been often assessed by the difference in an Euclidean space of vectors containing connectivity measures such as the clustering coefficient, shortest path length, degree distribution, etc. Here, we propose a radically different framework for studying brain connectivity differences. Instead of extracting a vector of features for each network (anatomical or functional), we jointly embed all of them in a common metric space that allow straightforward comparisons. Before embedding the functional and the anatomical networks into the common metric space, we aggregate a group of subjects (e.g., functional networks) according to Simas et al., (submitted) to obtain a group representation network. The method employed in this work allows to preserve connected components and to identify, among different subjects, a common underlying network structure. Our approach may provide a useful insight for the analysis of multiple networks obtained from multiple brain modalities or groups (healthy volunteers vs. patients, for instance).

## 2. Methods and materials

### 2.1. fMRI and DTI data

In this study we consider anatomical and functional brain connectivities-extracted from diffusion-weighted DW-MRI and fMRI data, respectively- defined on the same brain regions. Brain images were partitioned into the 90 anatomical regions (*N* = 90 nodes of the networks) of the Tzourio-Mazoyer brain atlas (Tzourio-Mazoyer et al., 2002) using the automated anatomical labeling method.

The anatomical connectivity network is based on the connectivity matrix obtained by Diffusion Magnetic Resonance Imaging (DW-MRI) data from 20 healthy participants, as described in Iturria-Medina et al. (2008). The elements of this matrix represent the probabilities of connection between the 90 brain regions of interest. These probabilities are proportional to the density of axonal fibers between different areas, so each element of the matrix represents an approximation of the connection strength between the corresponding pair of brain regions.

The functional brain connectivity was extracted from BOLD fMRI resting state recordings obtained as described in Valencia et al. (2009). All acquired brain volumes were corrected for motion and differences in slice acquisition times using the SPM5^1^ software package. All fMRI data sets (segments of 5 min recorded from healthy subjects) were co-registered to the anatomical data set and normalized to the standard MNI (Montreal Neurological Institute) template image, to allow comparisons between subjects. As for DW-MRI data, normalized and corrected functional scans were sub-sampled to the anatomical labeled template of the human brain (Tzourio-Mazoyer et al., 2002). Regional time series were estimated for each individual by averaging the fMRI time series over all voxels in each of the 90 regions. To eliminate low frequency noise (e.g., slow scanner drifts) and higher frequency artifacts from cardiac and respiratory oscillations, time-series were digitally filtered with a finite impulse response (FIR) filter with zero-phase distortion (bandwidth 0.01—0.1 Hz) as in Valencia et al. (2009).

A functional link between two time series *x*_*i*_(*t*) and *x*_*j*_(*t*) (normalized to zero mean and unit variance) was defined by means of the linear cross-correlation coefficient computed as *r*_*ij*_ = 〈*x*_*i*_(*t*)*x*_*j*_(*t*)〉, where 〈·〉 denotes the temporal average. For the sake of simplicity, we only considered here correlations at zero lag. To determine the probability that correlation values are significantly higher than what is expected from independent time series, *r*_*ij*_(0) values (denoted *r*_*ij*_) were firstly variance-stabilized by applying the Fisher's Z transform.

(1) $$Z_{ij}=0.5\ln\left(\frac{1+r_{ij}}{1-r_{ij}}\right)$$

Under the hypothesis of independence, *Z_ij_* has a normal distribution with expected value 0 and variance 1/(*df_ij_*−3), where *df* is the effective number of degrees of freedom (Bartlett, 1946; Bayley and Hammersley, 1946; Jenkins and Watts, 1968). If the time series consist of independent measurements, *df_ij_* simply equals the sample size, *N*. Nevertheless, autocorrelated time series do not meet the assumption of independence required by the standard significance test, yielding a greater Type I error (Bartlett, 1946; Bayley and Hammersley, 1946; Jenkins and Watts, 1968). In presence of auto-correlated time series *df* must be corrected by the following approximation $\frac{1}{df_{ij}}\approx \frac{1}{N}+\frac{2}{N}\sum_{\tau }\frac{N-\tau }{N}r_{ii}(\tau )r_{jj}(\tau )$, where *r_xx_*(τ) is the autocorrelation of signal *x* at lag τ.

### 2.2. Networks normalization

From Equation (1) our networks weights are in a non-normalized interval *Z_ij_* ∈ [*a*,*b*] ⊂ ℝ. In order to apply the framework described in Simas and Rocha (2015), we normalize our networks weights into the unit interval *I* = [0, 1] by means of a unique linear function:

(2) $$w_{ij}=\frac{\left(1-2\epsilon )Z_{ij}+(2\epsilon -1)\cdot MIN(Z_{ij}\right)}{MAX(Z_{ij})-MIN(Z_{ij})}+\epsilon$$

where ϵ in general is set to 0.01 in order to avoid merging and isolate vertices with weights at the boundaries of *Z_ij_* ∈ [*a*,*b*]. As proved in Simas and Rocha (2015), since the normalization is done by a unique linear function this does not affect networks properties.

### 2.3. fMRI networks aggregation and embedding

Among many ways to aggregate a group of networks here we employ a topological algebraic way to aggregate a group of networks. The networks group possess the same nodes but different edges values and can mathematical be represented by a weighted graph *G* = (*N*,*E*). *N* is the set of nodes representing the brain ROI's (*N* = 90 in this study) and *E* is the set of edges values (connections) between ROI's, e.g., ∀*e*_*i*,*j*_ ∈ *E*: *e*_*i*,*j*_ ∈ [0, 1] in the proximity space or ∀*d*_*i*,*j*_ ∈ *E*: *d*_*i*,*j*_ ∈ ℝ^+^_0_ ∪ {0, + ∞} in the distance space.

For the sake of simplicity, we denote a network with the same notation we use for the set of nodes *N*, i.e., a set of *n* networks (e.g., group of subjects) is represented by {*N*_*k*_} with *k*∈ {1, 2, 3.…, *n*}.

One possible way to aggregate a group of *n* networks is simply by averaging the homologous edges values. Obtaining in this way a group representative network, N^*^.

(3) $$N_{i,j}^{*}=e_{i,j}^{*}=\frac{\sum_{k\;=\;1}^{n}e_{i,j}^{\left[k\right]}}{n}$$

where *e*^[*k*]^_*i*,*j*_ is the edge *e*_*i*,*j*_ from network *N*_*k*_.

**Figure 1:**
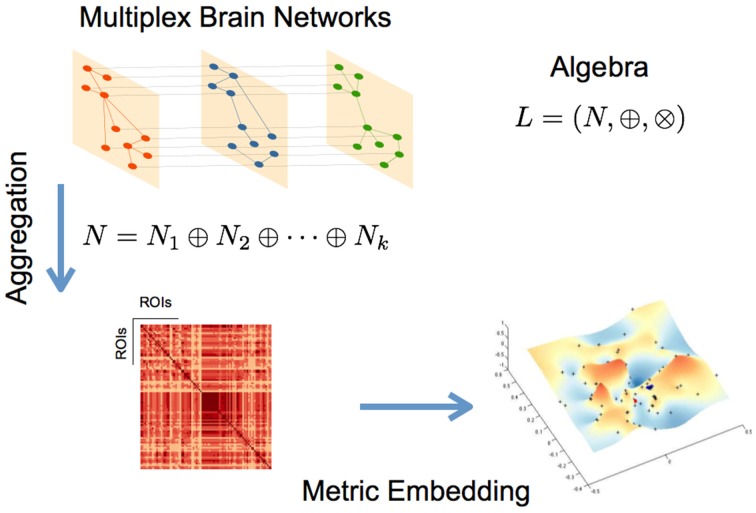
**Schematic representation of the main steps for the described networks aggregation and metric embedding (defined here for the algebra *L*)**.

Another way to aggregate networks, as explained in Simas et al., (submitted), is by considering all networks as a *multilayer* network (often called *multiplex*), which can be represented as a fourth-order tensor Simas et al., (submitted). This tensor can be represented as a extended matrix (Sole-Ribalta et al., 2013). The work of Simas and Rocha (2015), introduces a framework to aggregate networks in an algebraic way, relating it with fuzzy logic reasoning, and in Simas et al., (submitted) this work was extended to multilayer networks. In order to work algebraically with networks we have to set an algebra (defined as a vector space equipped with a bilinear product). This algebra allows us to perform algebraic operations with networks in the same way we perform algebraic operations in other contexts with other algebras (such as adding and multiplying real numbers). In short, a network can be represented by an adjacency matrix and a multilayer network by a tensor. Considering a set of tensors working under the algebra *L* = (*I*, ⊕, ⊗), where the weights (tensor entries) of the tensors in *I* ⊆ !!overlineℝ (subset of *extended real line*) and ⊕ and ⊗ two binary operators, we can represent a multilayer network with tensor *T* in this algebra. In Simas et al., (submitted) we have shown the particular case of multiplex networks, where layers are connected with weights *w*_*i*_,*i*,*L*_*k*_,*L*_*j*_ = 1 (in the proximity space), that the representative group network (e.g., functional) can be represented by N^*^ in the distance space (see below and Equation 6), as:

(4) $$N^{*}\equiv N_{1}⊕N_{2}⊕\ldots ⊕N_{k}$$

and the respective embedding by the following equation:

(5) $$N_{embedded}=N^{*}⊕N^{*2}⊕\ldots ⊕N^{*r}$$

where N^*^ is defined in Equation (4) and *r*, is the convergence parameter (Simas and Rocha, 2015; Simas et al., submitted). Figure 1 summarizes the metric embedding of a multiplex network described above.

Embedding a network of networks or, in our specific case, a multiplex fMRI network, allows us to determine which edges in the several layers contribute to the aggregation. We can therefore determine the subjects that contribute more/less or none to the aggregated network, and identify in each subject the sub-graphs for which they may have the highest contribution.

For our particular case, we embed our networks using the Metric Closure (Simas and Rocha, 2015) defined by the algebra *L* = ([ℝ^+^_0_ ∪ {+∞}, *min*, +), where ⊕ = *min* and ⊗ = +. The metric closure or metric embedding of a given network into a metric space, is a generalization of All Pairs Shortest Paths Problem (APSP) as shown in Simas and Rocha (2015). In this case the Johnson algorithm can be used to calculate the metric closure (Johnson, 1977).

Note that to calculate the metric closure (based on the Johnson algorithm) of a network we have to translate our networks from a proximity space into a distance space. There are many possible mappings to map a similarity space into a distance space, see (Simas and Rocha, 2015). Applying Equation (6) to all network weights, *w*_*i*,*j*_ ∈ [0, 1] (for more details see Simas and Rocha, 2015), we obtain the isomorphic distance network with weights *d*_*i*,*j*_ ∈ ℝ^+^_0_ ∪ {+∞}.

(6) $$d_{i,j}=\frac{1}{w_{i,j}}-1$$

The formalism behind the metric closure should not be confused with the formalism in Tropical Algebra geometry (Pachter and Sturmfels, 2004; Theobald, 2006). Both formalisms employ the same algebra for the isomorphism *d*_*i*,*j*_ = φ(*x*) = −*log*(*x*), which corresponds to a Schweizer-Sklar or Frank t-norm generator with λ =0 or 1 (see Equation 6 and Simas and Rocha, 2015) under the formalism in Simas and Rocha (2015). The formalism in Simas and Rocha (2015) uses any isomorphism φ to set a specific metric into a weighted graph when translated to the isomorphic distance space. A more detailed discussion on this relation between the work (Simas and Rocha, 2015) and Tropical geometry can be found in Simas et al., (submitted).

Embedding networks or multilayer networks allows us: (a) to detected clusters of nodes in a high-dimensional topological spaces, and by projecting the algebraic high-dimensional embedding into 3D, (b) it allows to perform exploratory networks analysis (c) to preserve the multilayer sub-structures across layers/subjects, better than other aggregations methods, as compared with the specific case of “simple” averaging (Equation 3).

Next we compare both methods of aggregation, “simple” averaging (Equation 5) and algebraic aggregation (Equation 5 according to Simas et al., submitted) of our fMRI networks, respectively, using our proposed method of embedding and comparing networks.

### 2.4. Multimodal networks comparison

In general, networks have been compared using statistical measures of local and global properties, such as: clustering coefficient, small-worldness, degree distributions, etc. We can find in the literature some examples of such techniques to compare multiple networks (Bullmore and Sporns, 2009; Stam, 2014). Our approach in this work is different. After embedding networks into the same metric space defined by the applied algebra, in our case *L* = (ℝ^+^_0_ ∪ {+ ∞}, *min*, +), we are able to compared them topologically. However, since networks generally come from different modalities (e.g., fMRI and DTI) it requires a previous step. We need to normalize the embedded edge weights distributions from the different modalities to the same average and variance to remove scale factors. One possible way to normalize both distributions, if we assume normality, is by calculating the z-score of the edge weights distributions (zero average and standard deviation set to the unit).

The embedded networks represent a hyper-grid in a multi-dimensional space with dimension equal or below to the number of nodes. In order to simplify and have some visual insight we can downgrade linearly this multidimensional grid into a 3D grid. This can be achieved applying to the embedded networks any technique for dimensionality reduction such as linear/non-linear Multi-Dimensional Scaling (MDS). MDS procedures refer to a set of related ordination techniques used in information visualization, in particular to display the information contained in a distance matrix (Borg and Groenen, 2005). These techniques guarantee, with a given distortion, that the relative distance between nodes is preserved in both multi-dimensional and low-dimensional reduction space. Plotting this low-dimensional grid (e.g., in 3D) we can use any statistical technique to fit a continuous surface into the data (see below **Figures 3, 4**). Its is natural to think that the difference between two surfaces obtained from different networks will emphasize topologically differences between the two connectivities. In this work we performed this operation in the multi-dimensional space by subtracting homologous embedded edges weights and take the absolute value of both embedded hyper-grids. This is, we subtract homologous embedded edges pairwise according to the formula:

(7) $$\begin{array}{l} M=|M_{fMRI^{*}}-M_{DTI}|\equiv \;\\ \;\{e_{i,j}^{diff}:\forall e_{i,j}^{*}\in M_{fMRI^{*}}\wedge \forall e_{i,j}^{**}\in M_{DTI}:e_{i,j}^{diff}=|e_{i,j}^{*}-e_{i,j}^{**}|\} \end{array}$$

*M* is the difference grid in the multi-dimensional space. Because the *M*-grid represents the difference between the two grids from different modalities (see above), the relative distance between nodes in *M* (given by Equation 7) should be concentrated at the origin if they are topological similar, otherwise widely distributed in the multi-dimensional space. Nodes at a distance from the origin of *s*-standard deviations are statistical different. Moreover, since we z-scored both embedded edge distributions this give us some degree of statistical significance when we compare both networks. All nodes that lay outside of a hyper-sphere with center at the origin with radius *R* = *s*, are statistical different. Here we had set σ = 1 for both distributions (z-score variables are estimated from the distributions of the embedded weights).

**Figure 2:**
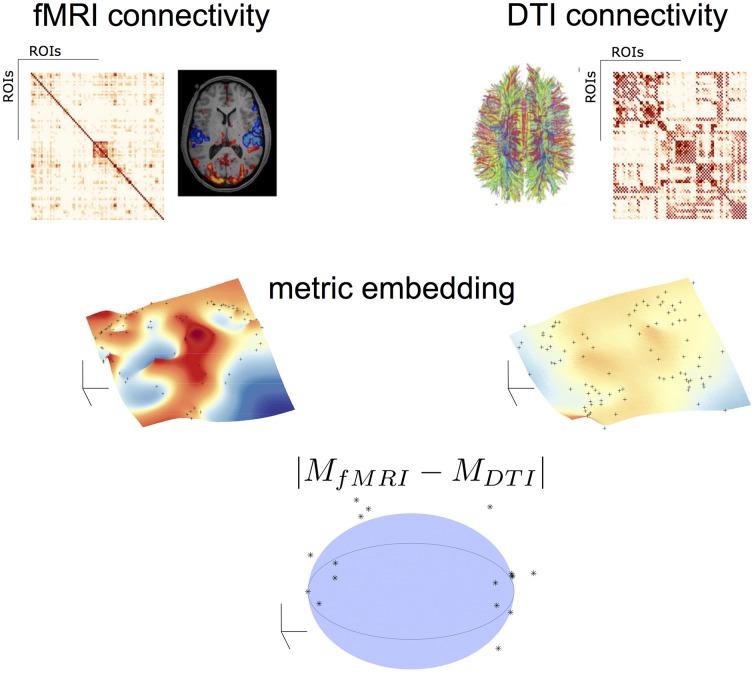
**Topological algebraic networks comparison**. Connectivity from different modalities (here fMRI and DTI) are firstly embedded (black dot points on the manifolds indicate the brain nodes) and then compared in a low-dimensional space. Black points outside the sphere correspond to nodes with a topological difference (at a given threshold) in the two modalities.

Figure 2 illustrates this process. After applying to both fMRI^*^ and DTI networks the same algebra and the metric embedding described above, both networks rely on the same metric space, therefore comparable. Topological differences can be visually seen in a linearly downgraded to 3D dimensions using a MDS technique, which preserves the relative distance between points in the grid (nodes or brain areas).

## 3. Results

**Figure 3:**
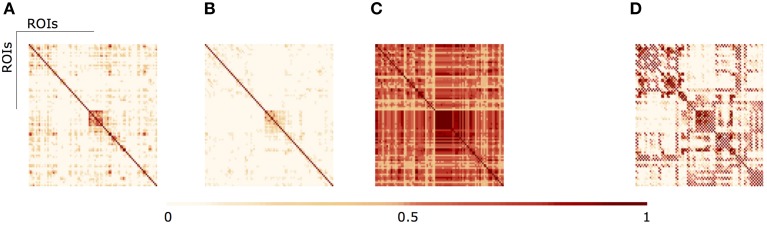
**(A)** fMRI single subject network **(B)** Average aggregated fMRI network **(C)** fMRI Algebraic Topologically aggregated (multiplex) network **(D)** DTI network.

In Figure 3 we illustrate the results of different aggregation procedures on the ensemble of fMRI networks. Compared with a fMRI connectivity matrix from a single subject (Figure 3A), one can notice the difference of a single averaging across subjects (Figure 3B) and our proposed algebraic topologically aggregated connectivity network (Figure 3C). It is clear that the averaging procedure tends to blur connectivity values between nodes. In contrast, the topologically algebraic aggregation can preserve components that are common across subjects. As other multilinear algebra or tensor-based analysis, our approach provide a natural mathematical framework for studying connectivity data with multidimensional structure. For illustrative purposes, we also show the DTI connectivity matrix in Figure 3D). It worths noticing the similarity of the anatomical connectivity structure with the aggregated (multiplex) connectivity obtained in Figure 3C. Moreover, since each layer encodes the functional network for a given subject, each subject contributes to the tensor aggregation/embedding with some or none connections (edges), as depicted in metric closure, Equation (5). If a layer do not contribute for the aggregation/embedding, we may consider this layer (subject network) as an outlier. Moreover, we are also able to identify the specific sub-network contribution (edges) of a given layer to the aggregation/embedding.

**Figure 4:**
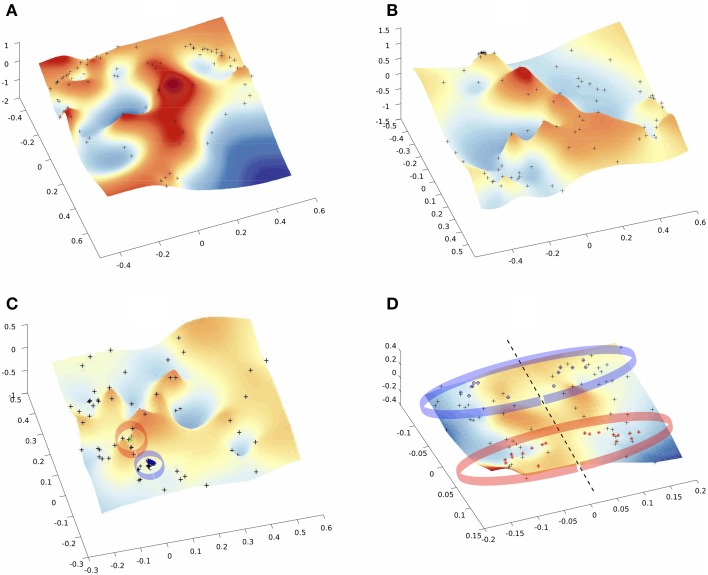
**Multi-Dimentional Scaling (MDS) of the embedded networks (A) fMRI single subject (B) fMRI average embedded network (C) fMRI Algebraic Topological aggregation (multiplex) embedded network (D) DTI embedded network**. Black dots indicate the embedded nodes. In plots **(C,D)**, blue and red points indicate the groups of brain areas discussed in the text.

Low-dimensional embeddings of different aggregated networks are illustrated in Figure 4. High-dimensional data, such as the information contained in the distance matrix obtained for the different networks, can be difficult to interpret. Here, multidimensional scaling (MDS) was used for visualizing the level of similarity of individual nodes of each -aggregated- network. The MDS algorithm aims to place each node in a low dimensional space such that the between-nodes distances are preserved as much as possible. This representation into a low-dimensional space enables an exploratory analysis and makes data analysis algorithms more efficient. Indeed, from the different plots of Figure 4 one can identify brain areas that are topologically close in the aggregated network as those points that are close on the 3D grid. This is clearly illustrated by the MDS representation of the multiplex functional network (Figure 4C). Nodes from the occipital regions form a compact group of nodes topologically close (with similar connectivity structure), as revealed by the blue points depicted on Figure 4C. We also notice that a compact group of nodes is formed by regions of the temporal lobe, putamen and insula, which are indicated by the red circle. Similarly, the anatomical network in Figure 4D clearly displays a natural organization, i.e., nodes of the two hemispheres lie on both sides of the dotted black line. Further, nodes from occipital regions in the anatomical network, indicated by the blue circles (including calcarina, cuneus, precuneus, …), are distantly located from the group of frontal brain areas indicated by the red marks.

**Figure 5:**
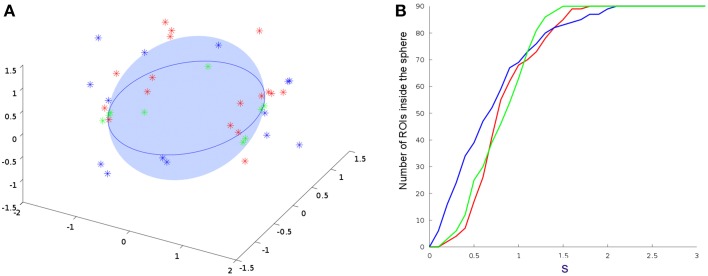
**Comparisons between DTI and all other embedded fMRI networks. (A)** 3D projections from Equation (7). Only points outside the sphere are plotted. **(B)** Number of ROI's inside the sphere of radius of *s*. Results from a single subject, average connectivity and multiplex networks are represented by the red, blue, and green points and curves, respectively. We consider the regions statistically different for *s*>1 and statistically equal for *s*<1. This shows that the multiplex algebraic aggregation (green) is more similar algebraically to DTI then average aggregation (blue) and single subject fMRI network (red).

Finally, Figure 5 displays the difference grid *M* in a low-dimensional space. As defined in Equation (7), *M* corresponds to the relative distance between nodes in networks from different modalities. Differences between brain areas are represented as points widely distributed in the low-dimensional space. Those nodes from different modalities (fMRI and DTI) that share an identical topological structure are located at the origin. The larger the difference in the connectivity structure, the larger the distance from the origin. By setting a threshold *s*, one can identify brain areas with similar connectivity as those points that lie inside of the hyper-sphere of radius *s* with center at the origin.

The number of brain regions (ROIs) with similar anatomical and functional connectivity are given in Figure 5B as a function of the threshold *s*. Curves correspond to the number of regions inside a hyper-sphere of various radius. We notice that the number of regions differ as a function of the aggregated network's type. It is worthy to mention that the differences above *s*-standard deviations are the important ones, since is above this threshold that the ROI's or nodes become statistical different when compare networks. In our example, the fluctuations below one standard deviations may give us some trend but all nodes in the networks are statistical equal for all types of aggregation. For our specific case, as an example, the brain areas located outside the hyper-sphere of radius *s* = 1.2 for the two types of aggregation, are listed in the Table 1.

**Table 1:** **ROIs with connectivity differences from DTI at 1.2 standard deviation**.

**AVERAGED AGGREGATED NETWORK**	
Calcarine (left)	Superior occipital gyrus (left)
Calcarine (right)	Superior occipital gyrus (right)
Cuneus (left)	Middle occipital gyrus (left)
Cuneus (right)	Middle occipital gyrus (right)
Inferior occipital gyrus	Insula (right)
Lingual (left)	Superior temporal gyrus (left)
Lingual (right)	Superior temporal gyrus (right)
**MULTIPLEX AGGREGATED NETWORK**	
Posterior cingulate gyrus (right)	Middle occipital gyrus (right)
Amygdala (right)	Inferior occipital gyrus (left)
Postcentral gyrus (right)	Inferior occipital gyrus (right)
Superior Temporal gyrus (right)	Thalamus (left)
	Heschl (right)

## 4. Discussion

The recent prevalence of applications involving multidimensional and multimodal brain data has increased the demand for technical developments in the analysis of such complex data. Indeed, the discrepancy between structural and functional brain connectivity is a current challenge for understanding general brain functioning. In this paper, we presented a method for characterizing the correspondences between functional and anatomical connectivity. To summarize, the main steps of our method are:

1. Metric network embedding: This procedure embed a group of connectivity graphs in a common space allowing straightforward comparisons. In contrast with simple averaging of connectivity matrices, the topologically algebraic aggregation can preserve components that are common across different subjects or different neuroimaging modalities. This tensor-based aggregation allows enhancing the common underlying structures providing a natural mathematical framework for studying connectivity data with multidimensional structure.
2. Multimodal Networks comparison: the differences between the embedded networks are calculated and represented in a low-dimensional space. Multi-Dimensional Scaling simply enables to display the information contained in the resulting distance matrix allowing thus an exploratory analysis of the data.
3. Detection of nodes (ROI's) with different connectivities: from points widely distributed in the low-dimensional space one can detect brain nodes that share a similar topological structure as those points are located close to the the origin. One can identify brain areas with the largest difference between anatomical and functional connectivity as those points located outside an imaginary hyper-sphere of a radius given by a threshold (Table 1)

Our findings suggest that embedding a brain network on a metric space may reveal regions that are members of large areas or subsystems rather than regions with a specific role in information processing. This is clearly illustrated for the anatomical network in Figure 5D, where frontal and occipital brain areas of both hemispheres are situated at distantly and located points of the space. Contrary to a classical averaging of connectivity matrices, the embedding of the multiplex functional network reveals brain areas that play a role in large brain system such as the occipital regions, known to be active when the subject is at wakeful rest.

Although experimental evidence suggests that functionally linked brain regions have an underlying structural core, this relationship does not exhibit a simple one-to-one mapping (Wang et al., 2014). These correspondences have also been investigated in specific subsystems, must of them focused on the default mode network (DMN), which is a group of brain regions that preferentially activate when individuals engage in internal tasks, i.e., when the subject is not focused on the outside world but the brain is at wakeful rest. Several studies report that the DMN exhibits a high overlap in its structural and functional connectivity (Honey et al., 2009; Wang et al., 2014). Nevertheless, strong discrepancies have been reported and strong functional links can be found between regions without direct structural linkages (Honey et al., 2009).

At a group level, one of the reasons for this discrepancy between structural and functional connectivity has been suggested to be the functional variability across subjects (Skudlarski et al., 2008; Honey et al., 2009; Wang et al., 2014). Indeed, clinical studies have provided evidence for a large heterogeneity of the functional connectivity, particularly in groups of patients with brain disorders such as neuropsychiatric disorders, which strongly alters the structural-functional relationships (Wang et al., 2014). Analytical tools are therefore required to account for this variability in order to enhance the common underlying network structure.

Results suggest that averaged aggregation captures the general differences in regions that play a role in visual, auditory and body self-awareness processes, but fails to identify in detail other specific areas *across* the subjects/groups. In Table 1 we observed that the average aggregation essentially captures part of visual (calcarine, cuneus, lingual, occipital), auditory (superior temporal gyrus), and insula regions that are associated to visual process and body self-awareness. Detection of visual and auditory regions suggest that the averaged aggregation mainly capture regions activated by the resting state condition of the recordings.

From the multiplex aggregation (or algebraic aggregation) shown in Table 1, we observed that besides capturing the well-known visual (occipital areas), primary sensory cortex (postcentral), and auditory regions (Heschl gyrus, superior temporal, thalamus), this approach also captures some other network sub-structures involved in touch activation (postcentral gyrus, thalamus) and emotional state activations (amygdala, thalamus, posterior cingulate). This alines with our claim that algebraic aggregation preserves better the multilayer sub-structures across a group of subjects (multilayers) accounting for as much of the variability in the data as possible.

Although we cannot definitively provide a one-to-one mapping of the structural and functional connectivity, we think that our method could provide new insights on the organization of brain networks during diverse cognitive or pathological states. We therefore hope that our approach will foster more principled and successful analysis of multimodal brain connectivity datasets.

For all the methods described in this article we provide the corresponding MATLAB software code. Data and code are freely available at the website https://sites.google.com/site/fr2eborn/download.

### Conflict of interest statement

The authors declare that the research was conducted in the absence of any commercial or financial relationships that could be construed as a potential conflict of interest.

## Footnotes

^1^http://www.fil.ion.ucl.ac.uk
